# Functional variants of the melanocortin-4 receptor associated with the Odontoceti and Mysticeti suborders of cetaceans

**DOI:** 10.1038/s41598-017-05962-1

**Published:** 2017-07-18

**Authors:** Liyuan Zhao, Xiaofan Zhou, Antonis Rokas, Roger D. Cone

**Affiliations:** 1 Third Institute of Oceanography, SOA, Xiamen, 361005 China; 2 Department of Molecular Physiology and Biophysics, Vanderbilt University School of Medicine, Nashville, TN 37232 USA; 3 Department of Biological Sciences, Vanderbilt University, Nashville, TN 37235 USA; 4 Life Sciences Institute and Department of Molecular and Integrative Physiology, University of Michigan, Ann Arbor, MI 48105 USA; 5 Guangdong Province Key Laboratory of Microbial Signals and Disease Control, Integrative Microbiology Research Centre, College of Agriculture, South China Agricultural University, Guangzhou, China

## Abstract

Cetaceans, a group of mammals adapted to the aquatic environment that descended from terrestrial artiodactyls, exhibit tremendous interspecific differences in a number of phenotypes, including feeding behavior, such as filter feeding in the Mysticeti vs prey-hunting Odontoceti, and size, with the smallest cetacean, the vaquita, at 1.4 meters and the largest, the blue whale, reaching 33 meters. The Melanocortin-4 receptor (MC4R) regulates food intake, energy balance, and somatic growth in both mammals and teleosts. In this study, we examined allelic variants of the *MC4R* in cetaceans. We sequenced the *MC4R* from 20 cetaceans, and pharmacologically characterized 17 of these protein products. Results identified a single variation at amino acid 156 in the MC4R from representative species of major cetacean lineages uniquely associated with the toothed whales or Odontoceti (arginine at 156) and baleen whales or Mysticeti (glutamine at 156). The Q156 receptor variant found in the larger baleen whales was functionally less responsive to its endogenous anorexigenic ligand, α-MSH. Furthermore, the R156 receptor variant showed greater constitutive activity and a higher affinity for ligand. These data suggest that the *MC4R* may be one gene involved in the evolution of feeding ecology, energy balance, and body size in cetaceans.

## Introduction

Cetaceans, including whales, dolphins and porpoises, are mammals that have secondarily adapted to the marine environment, and are thought to have diverged from ancestors closely related to modern terrestrial artiodactyls approximately 56–53 million years ago (Ma)^1^. The ancient cetaceans (called archaeocetes) evolved through amphibious stages to become fully aquatic by 40 Ma^2^. The cetacean crown group evolved from archaeocetes at about 34 Ma and gradually conquered nearly all the oceans, from tropical to polar waters, and even some estuaries and rivers^3–5^. They evolved into two sister clades, the Mysticeti (baleen whales) and the Odontoceti (toothed whales), which jointly contain approximately 89 extant species in 14 families^6^. Because extant cetaceans differ dramatically in terms of morphology and ecology, their evolution has attracted marked public and scientific interest.

The two suborders of Cetacea exhibit many different characteristics. Baleen whales developed bulk filter feeding, engulfing large volumes of prey-laden water to obtain vast amounts of small swarming plankton at low trophic levels^7–9^. In contrast, toothed whales possess the ability for high-frequency echolocation and deep diving to hunt large single prey items. Their diets usually include fishes or squids or even large vertebrates (eg. killer whale) at high trophic levels^7, 8, 10^. In addition, most baleen whales also undertake long-distance seasonal migrations between highly productive feeding grounds in the summer and breeding grounds in the winter, and may thus be subject to a long fasting period^11, 12^. Cetaceans are also remarkably diverse in body size (Table 1), and this diversity is likely to have been an important component of their ecological diversification and adaptation to marine environments. The order Cetacea includes the largest living organism, the blue whale (Mysticeti, maximum length of approximately 33 m and weight of 190,000 kg), but also has some very small members such as the vaquita (Odontoceti, 1.4 m in length and weight less than 40 kg)^4^. It has been suggested that the differences in body size in cetaceans are related to dietary specialization, which may have played a significant role in cetacean evolutionary history^10^. The unusually large size of some cetaceans has been explained by a macroevolutionary tradeoff mechanism^13^. The rate of body mass evolution in cetaceans was found to be higher than any terrestrial mammalian order, and this may be attributable to the reduced constraint on body mass of cetaceans following the transition into the ocean^14^. Some researchers also reconstructed the body size in extinct cetaceans to further understand the evolutionary trend in body size^15, 16^. However, with limited exceptions^17^, there have been very few studies of the potential molecular control mechanisms underlying the divergent size and feeding behaviors in cetaceans.

**Table 1:** Estimated body length and weight of cetaceans used in this study.

species	common name	suborder	Length (m)	Weight (t)	aa156 substitution
*Eubalaena glacialis*	North Atlantic right whale	Mysticeti	13.70	55.00	Q
*Caperea marginata*	Pygmy right whale	Mysticeti	6.21	3.25	Q
*Eschrichtius robustus*	Gray whale	Mysticeti	14.63	30.00	Q
*Balaenoptera musculus*	Blue whale	Mysticeti	33.58	110.00	Q
*Balaenoptera physalus*	Fin whale	Mysticeti	21.20	33.22	Q
*Balaenoptera borealis*	Sei whale	Mysticeti	16.09	20.00	Q
*Megaptera novaeangliae*	Humpback whale	Mysticeti	17.98	39.31	Q
*Physeter macrocephalus*	Sperm whale	Odontoceti	11.03	37.42	R
*Berardius arnuxii*	Arnoux’s beaked whale	Odontoceti	8.85	8.50	R
*Mesoplodon europaeus	Gervais’ beaked whale	Odontoceti	5.20	0.73	R
*Mesoplodon peruvianus	Pygmy beaked whale	Odontoceti	3.52	NA	R
*Inia geoffrensis*	Amazon river dolphin	Odontoceti	1.98	0.09	R
*Pontoporia blainvillei	La plata dolphin	Odontoceti	1.49	0.03	R
*Delphinapterus leucas*	Beluga whale	Odontoceti	3.81	0.64	R
*Orcinus orca	Killer whale	Odontoceti	7.92	2.05	R
*Neophocaena phocaenoides*	Finless porpoise	Odontoceti	1.41	0.03	R
*Phocoena phocoena*	Harbor porpoise	Odontoceti	1.86	0.06	R
*Pseudorca crassidens*	False killer whale	Odontoceti	5.06	0.58	R
*Feresa attenuata*	Pygmy killer whale	Odontoceti	2.43	0.14	R
*Tursiops truncatus*	Common bottlenose dolphin	Odontoceti	2.37	0.21	R
*Cephalorhynchus heavisidii*	Haviside’s dolphin	Odontoceti	1.69	0.07	R

The MC4R from species marked with “*” were not studied pharmacologically.

The central melanocortin system is one of the best-characterized central neural circuits involved in the regulation of energy homeostasis^18^. This collection of circuits is unique in that it has the capability of sensing signals from a wide array of hormones, nutrients, and afferent neural inputs. These circuits are involved in integrating long-term adipostatic signals from leptin and insulin, primarily received by the hypothalamus, with acute signals regulating hunger and satiety primarily received by the brainstem^19^. Furthermore, the system has been demonstrated not only to be involved in the regulation of long term energy homeostasis via the regulation of both feeding behavior and energy expenditure, but to be involved in somatic growth as well^20–22^.

In this system, the melanocortin-4 receptor (MC4R), a Gs-coupled receptor, is demonstrated to play a significant role in regulation of energy homeostasis in vertebrate species from teleosts to humans^23–28^. MC4R activation is known to decrease food intake and increase energy expenditure^26^. The receptor has a modest effect on somatic growth in mammals^24, 26^, and mutations in the MC4R are the most common cause of monogenic early-onset obesity in humans^24^. Though most of the research on the MC4R has been performed in mice and humans, MC4R has also been demonstrated to be highly conserved, even in early branching vertebrates such as fish^29^. Increased MC4R activity early in the development of zebrafish embryos appears to cause a decrease in growth^30^. Moreover, naturally occurring mutations in *MC4R* have a profound effect on the rate of maturation, fecundity, size, weight, mating and feeding behavior in platyfish^23^ and cavefish^31^. Based on the important role of the MC4R in feeding behavior, energy homeostasis and size in both fishes and mammals, we report here on the sequence and function of the *MC4R* in cetaceans. In this study, we sequenced the *MC4R* from 20 cetaceans, and performed pharmacological assays on 17 of these protein products *in vitro*. We describe an interesting variation uniquely associated with the two suborders in Cetacea, and characterize the pharmacological properties of these variant receptors.

## Results

### Characterization of *MC4R* genes in cetaceans

**Figure 1:**
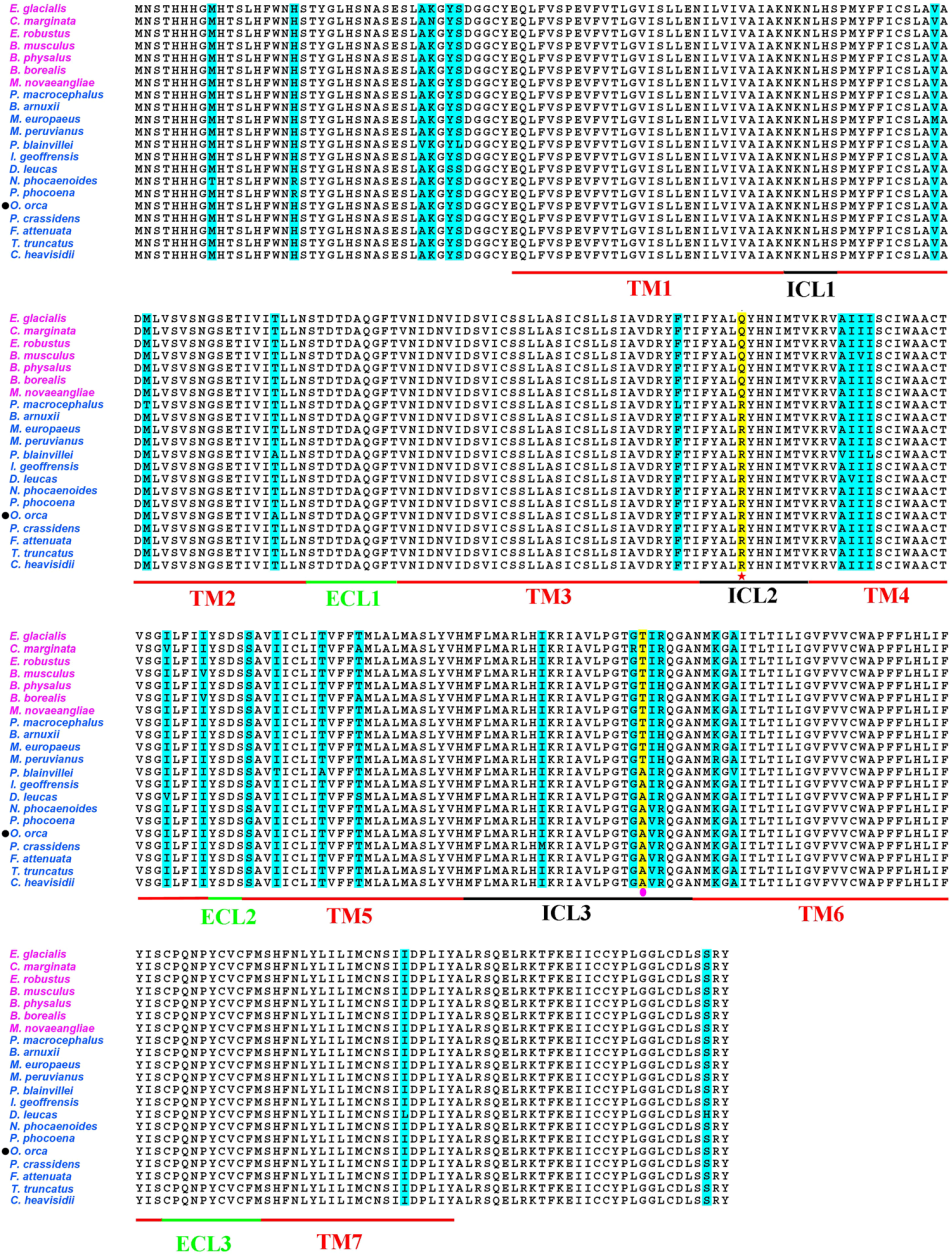
Sequence alignment of 21 cetacean MC4R orthologs. Residues that are variable across species are shaded, identical residues are without shadings. TM = transmembrane domain, ECL = extracellular loop and ICL = intracellular loop. Blue shading variants present only in a few (no more than four) species. Yellow shading variants present in many (14 and 10) species. Residue marked with a red star is position 156 located in ICL2. Residue marked with pink circle is position 234 located in ICL3. Species names with pink characters are mysticetes, with blue characters are odontocetes. *O. orca* sequence marked with a black circle was downloaded from Genbank.

Since high quality mRNA from cetaceans is difficult to obtain, and the *MC4R* is encoded by a single coding exon, we cloned *MC4R* genes from 20 cetacean species using genomic DNA. All 20 of the determined cetacean *MC4R* orthologs contain an open reading frame (ORF) of 999 nucleotides (nt) encoding a protein of 332 amino acid (aa) residues followed by a stop codon. We aligned all the deduced aa sequences of ORFs from 21 cetacean species including *O. orca*, which was available from GenBank (accession number: XP_004268107). These cetacean MC4R aa sequences showed high sequence similarity to other vertebrate MC4R orthologs, and exhibited a typical predicted MC4R domain architecture, including 7 transmembrane domains (TMs), 3 extracellular loops (ECLs) and 3 intracellular loops (ICLs) (Fig. 1).

**Figure 2:**
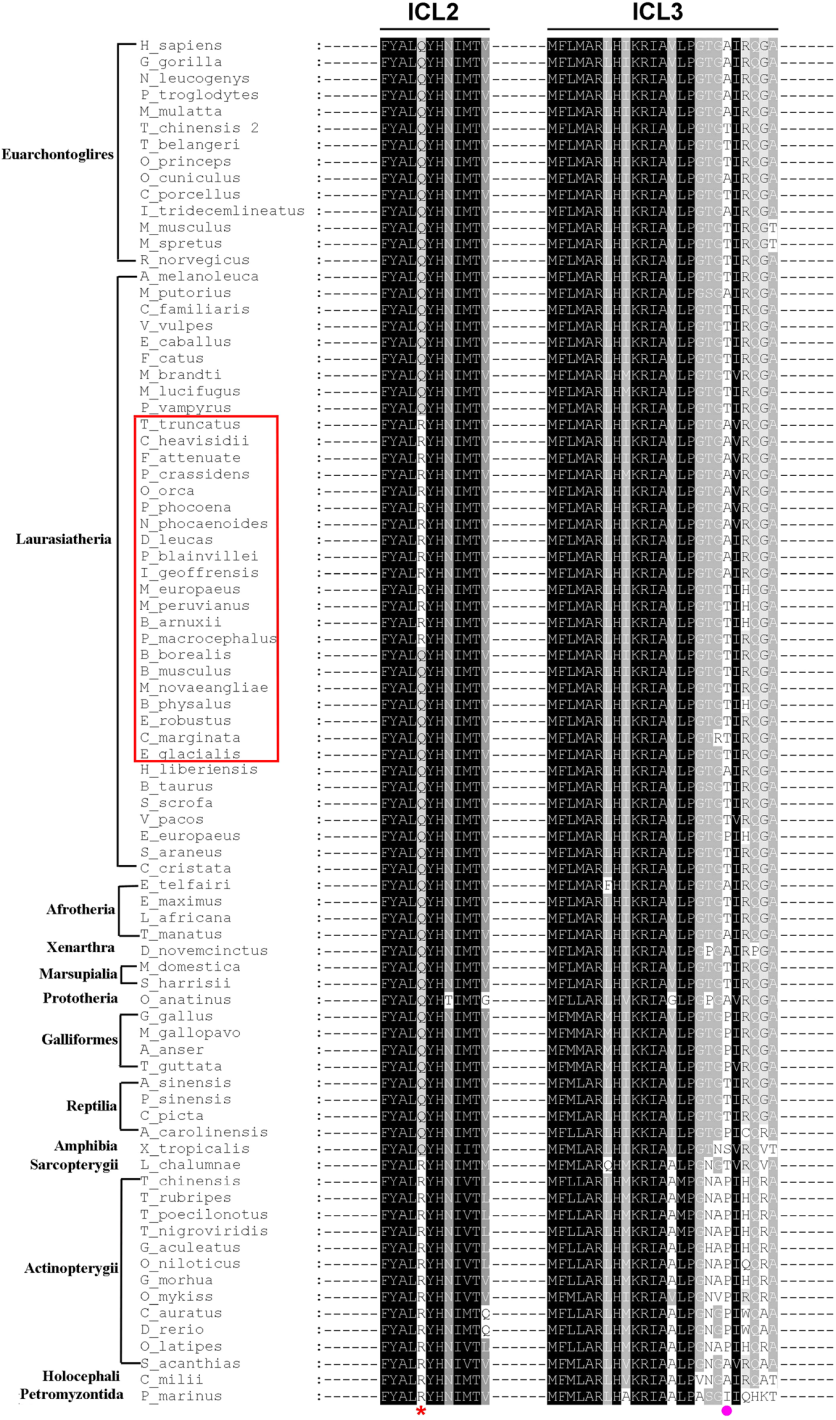
Amino acid sequence alignment of the ICL2 and ICL3 domain of MC4R in vertebrate orthologs. Black shading shows identical amino acid residues, gray shading shows similar amino acid residues, and unshaded regions indicate dissimilar residues. The species in the red square frame are the cetacean species. The red asterisk indicates the R/Q variant at position 156. The pink circle indicates the A/T variant at position 234.

Nevertheless, these sequences still had some interesting variants. Among these variants, most were only found in a few species, while two, at position 156 located in ICL2 and position 234 located in ICL3 respectively (amino acid positions are numbered according to the human MC4R sequence) were detected in many species. Interestingly, at position 156, two different residues were associated with the 2 different cetacean suborders. The MC4R sequences of all 7 mysticetes had a glutamine (Q) at position 156, while the 14 odontocete species examined all contained an arginine (R) (Fig. 1). We further examined more MC4R orthologs from public sequence databases. Interestingly, among the MC4R sequences reported, all fish have R at position 156, while all tetrapods, except odontocetes, have residue Q (Fig. 2).

### Phylogenetic and evolutionary analysis of *MC4R* sequence in cetaceans

**Figure 3:**
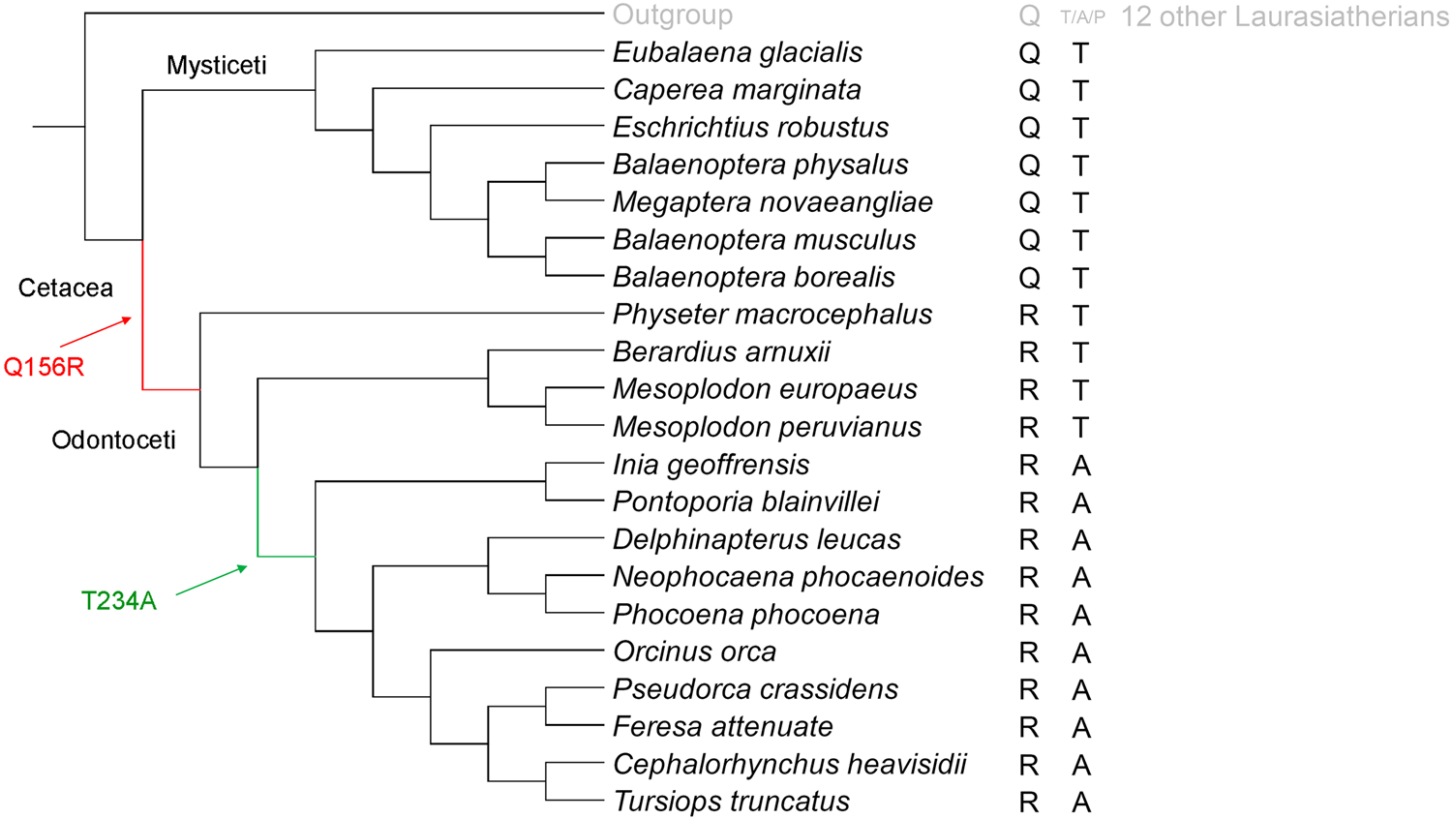
A cladogram showing the evolutionary history of the cetacean MC4R gene. The topology reflects the relationship between the 21cetacean species with 12 additional Laurasiatherian mammals included as outgroups. It is statistically equivalent with MC4R gene trees inferred with multiple approaches (see Methods). The amino acid residues at the site 156 and 234 of each MC4R gene and each species are listed on the right. The inferred substitution from Q to R that occurred in the most recent common ancestor of Odontoceti was highlighted in red, while the substitution from T to A was highlighted in green.

To understand how the sequence changes in the *MC4R* gene correlate with the evolution of Cetacea, we performed phylogenetic analyses of the *MC4R* genes in all the aforementioned 21 cetacean species with 12 other Laurasiatherian mammals as outgroups. Due to the high conservation of *MC4R* sequences, many internodes in the resulting gene trees were not resolved or only received marginal support regardless of the approaches (maximum-likelihood and Bayesian) or model types (nucleotide- and codon-based model) being used (supplementary Fig. 1). Nevertheless, the trees in general showed strong support for the monophyly of Mysticeti and Odontoceti, as well as several other well established clades such as Ziphiidae, Inioidea, and Delphinoidea. Our topology test further showed that the estimated *MC4R* gene trees were not significantly different from the tree reflecting the currently accepted relationships between cetaceans^3^ (Fig. 3; Supplementary Table S1). The results suggested that the evolutionary history of *MC4R* was congruent with that of Cetacea. We reconstructed ancestral *MC4R* sequences based on this Cetacea species phylogeny and inferred that a Q to R substitution at site 156 occurred in the most recent common ancestor (MRCA) of Odontoceti after its separation from Mysticeti, and a T to A substitution at site 234 occurred in the MRCA of Dephinida after its separation from Ziphiidae. However, our branch-site model analyses did not find evidence of positive selection (Supplementary Table S2).

### Characterization of the pharmacological properties of Q156 and R156 variants of the MC4R protein

MC4R is a G-protein coupled receptor (GPCR) that plays an important role in the regulation of food intake, energy balance, and body weight control^19^. Its constitutive or ligand-regulated activity can be assayed by measuring cAMP concentrations either directly, or indirectly by measurement of cAMP dependent reporter gene expression^32^. Further, the effect of receptor sequence variation on agonist and antagonist ligand binding can also be measured.

**Figure 4:**
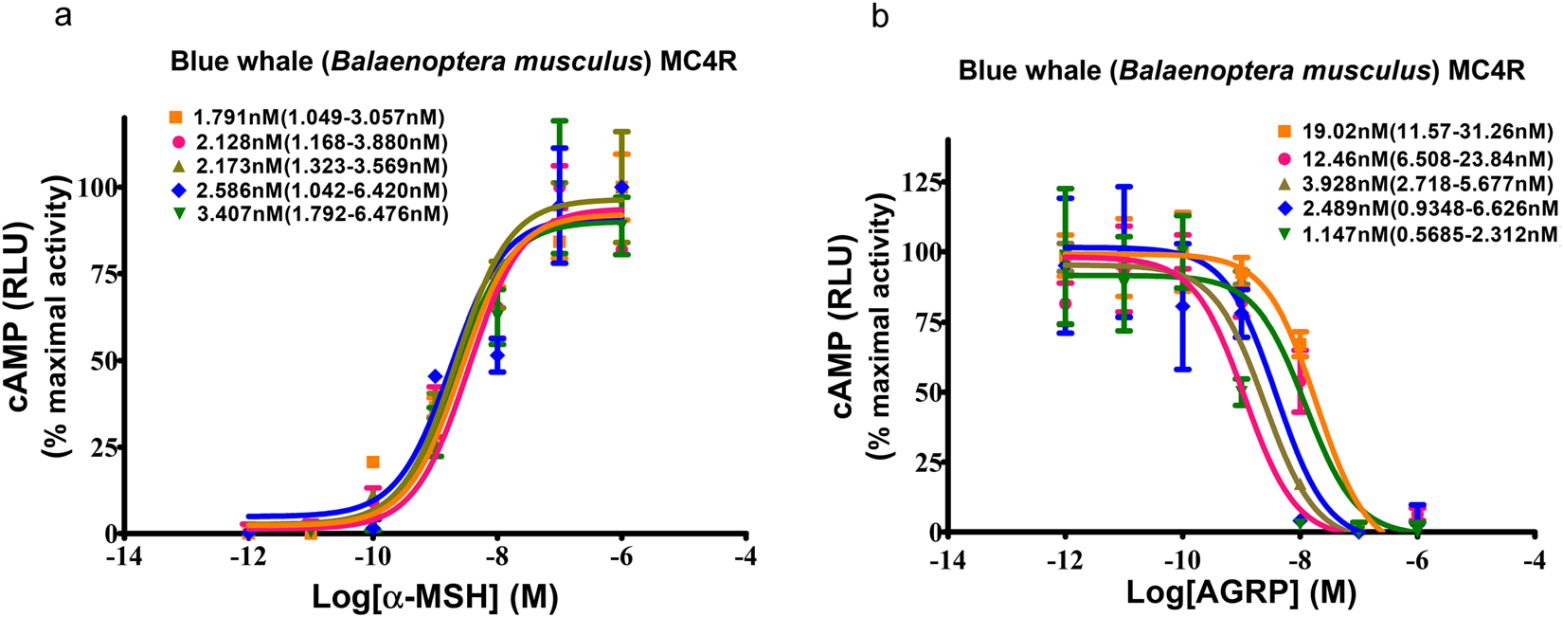
A cAMP assay used for characterization of cetacean MC4R orthologs. For functional characterization of cetacean MC4R orthologs, DNA were cloned into expression vector pcDNA3.1(+), then transiently transfected into HEK293T cells, and finally tested for agonist-induced cAMP accumulation. The cAMP assay was performed by measuring luciferase activity under the control of a cAMP response element promoter as described in Methods. Concentration-response curves were generated by cells stimulated with increasing amounts of α-MSH (**a**) or with α-MSH 10^−9^ M and increasing concentrations of AgRP (**b**). Data were normalized to maximal α-MSH stimulation after subtraction of basal activity and were fitted by nonlinear regression. For blue whale (*Balaenoptera musculus*) MC4R, five independent experiments were tested, each performed in triplicate. The estimate of the EC_50_ (**a**) or the IC_50_ (**b**) and their 95% confidence intervals for each independent experiment are shown. RLU, relative luminescence units.

Cetaceans are remarkably diverse in diet, feeding behavior and body size, with notable differences between the two extant suborders, Mysticeti and Odontoceti. Based on the sequence alignment of cetacean *MC4R*, we identified an interesting variation at position 156 uniquely associated with the two suborders in Cetacea, and thus we sought to compare MC4R EC_50_ values between mysticetes and odontocetes. Using the above approaches, we measured the ligand binding and functional response of all 17 different cetacean MC4Rs that we were able to successfully express in HEK293 cells by transfection. As shown in Fig. 4a (here we use blue whale, *Balaenoptera musculus*, as an example), the EC_50_ response to the native ligand, α-MSH, could be reproducibly determined by the dose response curve of MC4R to α-MSH being 2.4 ± 0.3 nM. The IC_50_ response to AgRP, the endogenous MC4R antagonist, could be similarly calculated, to be 7.8 ± 7.7 nM (Fig. 4b). The inter-assay variability in the EC_50_ response to α-MSH was minor; greater inter-assay variability in response to AgRP was noted.

**Figure 5:**
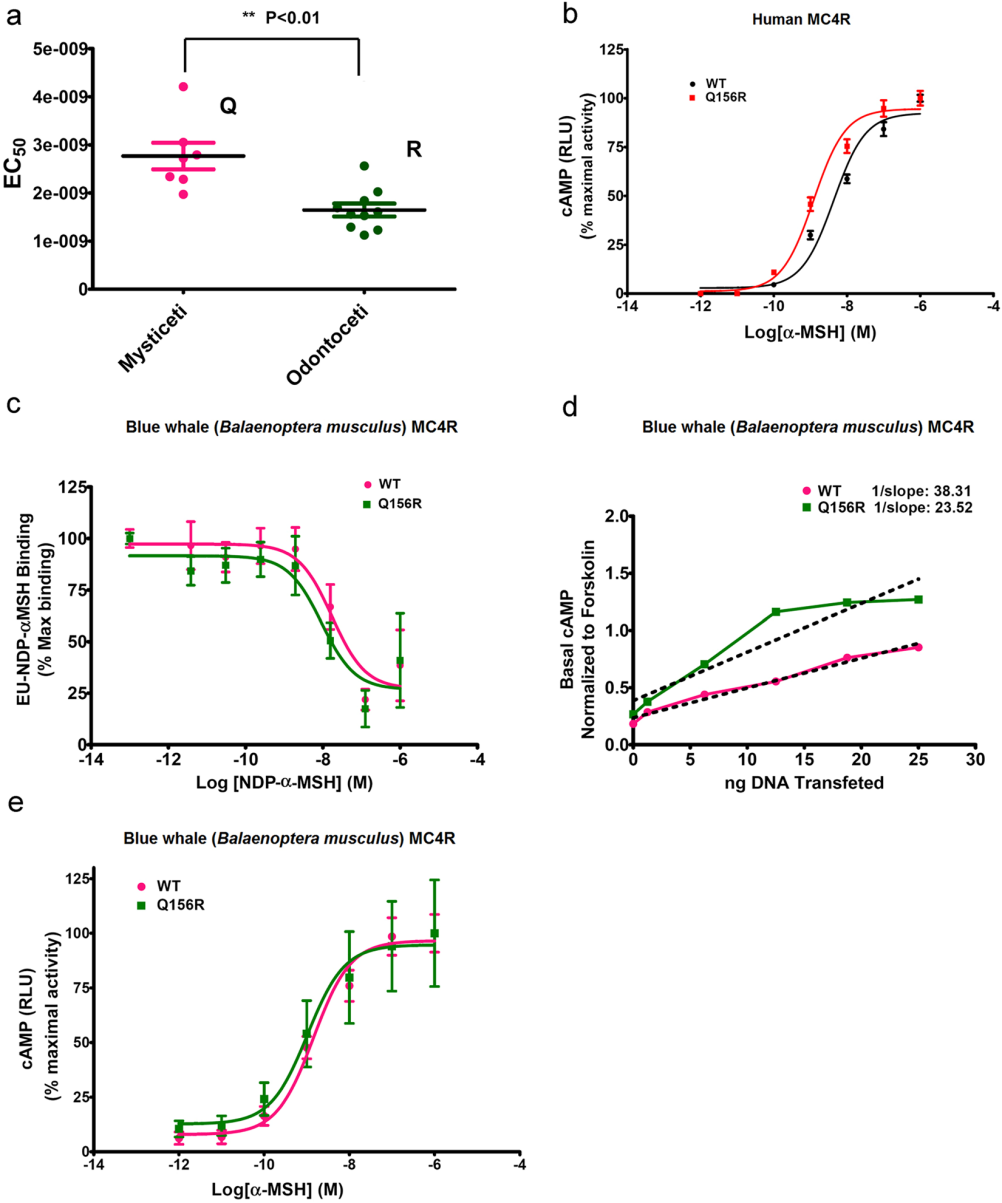
Functional characterization of mutations at position 156 in human and cetacean MC4R. (**a**) EC_50_ values of cetacean MC4R orthologs grouped into suborders, Mysticeti (Gln^156^) and Odontoceti (Arg^156^). EC_50_ values were determined from concentration-response curves from 3 independent experiments (*Methods*). Pink symbols are mysticetes; green are odontocetes. (**b**) cAMP production in HEK293T cells transiently transfected with wild-type hMC4R (black circle) or Q156R hMC4R (red square) in response to various concentrations of α-MSH. (**c**) Competitive binding assays in wild-type (pink circle) and Q156R variant (green square) of the blue whale (*B. musculus*) MC4R; unlabeled NDP-α-MSH used to displace the binding of EU-NDP-α-MSH. (**d**) Transient transfection experiment comparing basal activity levels of the wild-type (pink circle) and Q156R mutant (green square) of the blue whale MC4R upon increasing concentration of receptor plasmids. (**e**) cAMP production in HEK293T cells transiently transfected with wild type (pink circle) or Q156R mutant (green square) blue whale MC4R in response to various concentrations of α-MSH stimulation. (**b–e**) All data points were means ± SEM from three independent experiments, with triplicate wells in each experiment.

We then proceeded to test all 17 MC4R in the α-MSH EC_50_ assay (Table 1). In this test, we found the EC_50_ values of mysticete MC4R were significantly higher than odontocete MC4R (*p* < 0.01) (Fig. 5a). To probe the general role of this residue in MC4R function, we next generated a human MC4R mutant (Q156R), and compared its function with the wild-type MC4R. The EC_50_ value of WT hMC4R (containing residue Q^156^) was 3.3 times higher than the mutant (containing residue R^156^) (Fig. 5b, Table 2). This indicated that the introduction of an R residue at position 156 of the human receptor significantly increased the sensitivity of the receptor to its native ligand. Next, we generated a blue whale MC4R mutant (Q156R) and compared its function with WT receptor to investigate the function of residue 156 within the context of the blue whale MC4R. To determine residue 156 ligand binding affinity, we performed a competition binding assay using EU-NDP-α-MSH as a tracer. We found that introduction of R156 into the blue whale sequence increased binding affinity relative to the WT blue whale receptor construct (Fig. 5c, Table 2). In addition, we compared the basal activity of WT and MC4R mutant (Q156R) blue whale receptors, by examining the concentration-response effects of each receptor expression construct upon transfection in the absence of the ligand. The mutant Q156R receptor also exhibited higher constitutive activity relative to the WT receptor (Fig. 5d). Finally, examining EC_50_ curves for the wild type and Q156R mutant of the blue whale receptor, we observed a small, but still statistically significant, increase in functional ligand sensitivity in the Q156R blue whale receptor (Fig. 5e, Table 2).

**Table 2:** Functional characterization of selected MC4R orthologs or mutants.

Ortholog or mutant	Number of independent experiments	α-MSH-stimulated cAMP, EC_50_ (nM)	Eu-labeled NDP-α-MSH displacement binding, IC_50_ (nM)
Human WT	3	4.205 ± 0.9714	ND^a^
Human Q156R	3	1.283 ± 0.1242^b^	ND^a^
Blue whale WT	3	1.445 ± 0.0328	18.24 ± 3.565
Blue whale Q156R	3	1.058 ± 0.1318^b^	9.379 ± 1.515^b^

For functional characterization, HEK293T cells were transiently transfected with MC4R or their mutant constructs. cAMP assays were performed using α-MSH, and unlabeled NDP-α-MSH was used to displace Eu-labeled NDP-α-MSH in binding assays. EC_50_ values were determined from concentration-response curves of agonists (1pm to 1 μm) using GraphPad Prism; IC_50_ values were determined from concentration-response curves of agonists (0.1pm to 1 μm) using GraphPad Prism. The data were expressed as the mean ± SEM of three independent experiments for the MC4R orthologs or mutants. ^a^ND, not done. ^b^Significantly different from corresponding WT receptor, *p* < 0.05.

### Functional analysis of MC4R from diverse cetaceans

**Figure 6:**
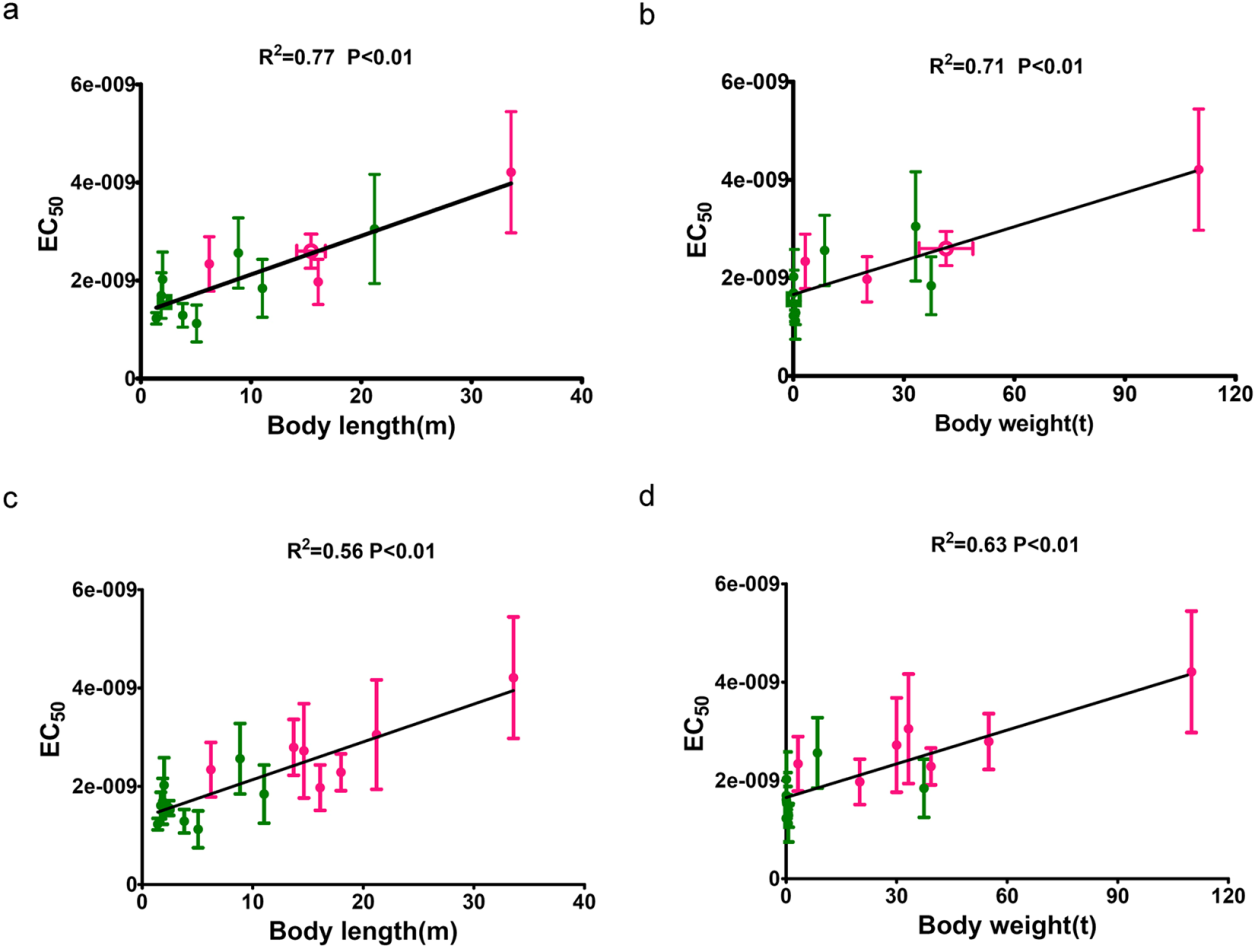
Association between MC4R activity and cetacean body size. EC_50_ values for response to the native ligand α-MSH were determined from concentration-response curves from 13 unique receptor amino acid sequences from 17 different species, as described in *Methods*. Each point represents data (mean ± SEM) from a unique receptor amino acid sequence, with circles representing unique sequences from individual species and hollow circles and hollow squares representing identical sequences shared by three species. All species were measured with three independent experiments in triplicate. ○: *Eubalaena glacialis*; *Eschrichtius robustus*; *Megaptera novaeangliae*, □: *Tursiops truncatus*; *Feresa attenuata*; *Cephalorhynchus heavisidii*. Pink symbols are mysticetes; green are odontocetes, vertical bars represent SEM of all EC_50_ values measured, horizontal bars represent SEM of length (**a**) and weight (**b**) in the points representing identical receptor sequences shared by multiple species. EC_50_ values for the native ligand α-MSH were determined from concentration-response curves from 17 individual cetacean species. Each point represents data (mean ± SEM) of EC_50_ values from three independent experiments. The correlations between EC_50_ value and phenotypic data (body length (**c**) and body weight (**d**)) were calculated using Bayes Traits v2.0 which properly account for the phylogenetic relationships between the organisms we sampled in this study.

Next, we analyzed the association between EC_50_ values of different cetaceans and their body size. We determined average EC_50_ values for all 17 cetacean receptors cloned into the same expression vector, repeating each EC_50_ curve 3 times. Not controlling for phylogenetic relatedness, and plotting identical receptor sequences from different species as single points, linear regression analysis of EC_50_ values with body length and body weight demonstrated that these values were correlated both with the cetacean body length and weight. The larger cetaceans had higher EC_50_ values, suggesting that these species had MC4R proteins with reduced sensitivity to the anorexigenic α-MSH ligand. *R* ^*2*^ for correlation between MC4R EC_50_ values and body length and body weight were 0.77 (*p* < 0.01) and 0.71 (*p* < 0.01) respectively (Fig. 6a,b). However, we did not find any differences in the responsiveness to AgRP among the different cetacean MC4Rs (Data not shown). When the data is controlled for phylogenetic relatedness, a significant association is still seen when all cetacean data is analyzed individually (Fig. 6c,d); *R* ^*2*^ for correlation between MC4R EC_50_ values and body length and body weight were 0.56 (*p* < 0.01) and 0.63 (*p* < 0.01) respectively.

## Discussion

Following their return to the marine environment, cetaceans evolved many features that differ dramatically from their mammalian predecessors. The variation in body size among cetaceans is far greater than that seen in any other extant mammalian order^14^. The largest living animal, the blue whale, is nearly 30 times larger than the African elephant^13^. The highly efficient bulk filter feeding allows blue whales to exploit large amount of prey at low trophic levels, and obtain the energy needed to main such an extreme body size^8, 9^. MC4R is well-known to be involved in the regulation of food intake, energy balance, and somatic growth in mice, humans, and zebrafish^19^. To survey whether MC4R variation was associated with cetacean feeding ecology, energy balance and body size, in this study we determined the sequence of 20 cetacean MC4Rs and characterized the pharmacological properties of 17 of these.

MC4R has been shown to be under strong purifying selection and subjected to high level functional constraint during the entirety of vertebrate evolution^33^. Our phylogenetic results suggested that the common ancestor of all cetaceans had a Q at site 156, similar to all extant land vertebrates, and the Q to R substitution occurred in the MRCA of odontocetes. Although we did not find evidence of positive selection on the branch leading to Odontoceti, this does not necessarily mean that the Q156R substitution was not adaptive; the vast majority of the *MC4R* gene was under very strong purifying selection^33^, and the test might not have enough power to detect positive selection at one site. It should also be noted that all statistical methods for detecting molecular adaptation, like the ones used in this study, are typically unable to identify adaptive evolution events that affect a single or a few amino acids^34^.

In this study, we reported the complete open reading frame (ORF) sequence of MC4R from 20 cetacean species. MC4R is a single-copy gene with a sufficiently high level of sequence divergence from its most similar homologs (MC3R and MC5R) to give us confidence that the 20 cetacean sequences we report here are MC4R orthologs. The MC4R coding sequence was found to be highly conserved between different cetacean species and was under strong purifying selection during cetacean evolution. Cetacean MC4Rs also showed high sequence similarity to MC4Rs in other vertebrates (Figs 1 and 2). Based on sequence comparison, two interesting amino acid differences can be seen in the cetacean MC4R protein sequences. First, we noted variant residues, Q or R, at position 156, that correlate perfectly with the Mysticeti (Q156) and Odontoceti (R156) suborders. Second, we also observed a T at position 234 in all mysticetes, and only 4 of the odontocetes (*P. macrocephalus*, *B.arnuxii*, *M. europaeus*, *M. peruvianus*). Intriguingly, these 4 odontocetes that have T at position 234 happen to be species with very large body sizes. It may be of interest to further examine the specific pharmacological effects of T234 given the general observation that larger cetaceans exhibited greater EC_50_ values, consistent with reduced sensitivity to the anorexic ligand α-MSH (Fig. 6).

The two suborders have very different feeding ecologies. Compared with odontocetes hunting single prey items at high trophic levels, the mysticetes use bulk filter feeding to acquire a large amount of prey at low trophic levels to obtain enough energy to maintain their extremely large body size. In this study, we focused on the Q156 and R156 variants, since these associated perfectly with the Mysticeti and Odontoceti suborders. Interestingly, we found that the EC_50_ values of mysticetes MC4R for activation by the native ligand α-MSH were significantly higher than odontocetes MC4R (Fig. 5a). To confirm a role for variation at position 156 in receptor function, we studied the pharmacological consequences of Q/R variation both in the context of the human and cetacean MC4R coding sequences. Introduction of an R in place of the WT Q residue at 156 in the human receptor significantly increased the receptor’s sensitivity to α-MSH (3.3-fold), as much as seen in the cetacean 156R variant. Next, we examined the MC4R of the biggest mysticete, the blue whale, which had the highest EC_50_ among all analyzed cetacean receptors. Introduction of an R at position 156 of this receptor increased apparent ligand affinity, increased receptor constitutive activity, and slightly, but significantly, increased α-MSH sensitivity. Thus, experiments in both the context of the human and cetacean receptor sequence support the hypothesis that an R at position 156 may serve to increase the basal and/or ligand mediated activity of the receptor.

Residue 156 and adjacent amino acids in the second intracellular loop have been previously studied in the human receptor, for example as a naturally occurring variant in the human population^35, 36^. The human Q156R variant does not appear to be associated with variations in BMI, but the mutation was reported to increase α-MSH responsivity, as indicated by a ~5 fold decrease in receptor EC_50_ values using α-MSH as agonist, in further support of the data reported here^36^.

Since MC4R has been demonstrated in multiple species to directly regulate feeding behavior^18, 19, 37^, the data on α-MSH responsivity shown here suggest that variation at position 156 of the MC4R could well have played some role in the evolution of divergent feeding behaviors in the mysticetes and odontocetes.

While MC4R appears to have consistent effects on food intake, its impact on somatic growth varies widely from species to species. Size variation within species is known to be highly polygenic and involve multiple physiological systems^38–40^ and genes, including GH, IGF1, leptin, and ubx^41–44^. While the Q/R variation at position 156 of the MC4R does not have a monogenic effect on size determination, given the existence of the Q156 allele of the receptor in mammals and the existence of the R156 allele in the larger odontocetes, we nonetheless observed that EC_50_ values for the 17 non-redundant cetacean MC4Rs were significantly correlated with both body length and body weight (Fig. 6) with the Q156 receptor variant in the larger cetaceans exhibiting less sensitivity to the anorexigenic native ligand, α-MSH. The amino acid variant observed in the four large odontocete species (234T) may also play some role in receptor function. No difference in response to the endogenous receptor antagonist AgRP was seen, however, in contrast to α-MSH, responsiveness to AgRP exhibited significant inter-assay variability, making it difficult to interpret data on AgRP. Thus, any role of the Q/R variants of the MC4R in size determination would likely be complex, multigenic, and indirect. For example, the association of Q/R allele variation with the general size variations seen between the odontoceti and mysticeti may be indirectly related to the effect of these alleles on feeding behavior.

## Methods

### Cetacean DNA samples and phenotypic data

DNA samples were provided by K. Robertson (SWFSC Marine Mammal and Turtle Molecular Research Sample Collection, Southwest Fisheries Science Center, NOAA, La Jolla, CA). There were 21 cetacean species examined in this study, *Eubalaena glacialis* (SWFSC Z13086)*, Caperea marginata* (SWFSC Z5989), *Eschrichtius robustus* (SWFSC Z52434), *Balaenoptera musculus* (SWFSC Z124030), *Balaenoptera physalus* (SWFSC Z14336), *Balaenoptera borealis* (SWFSC Z30480), *Megaptera novaeangliae* (SWFSC Z61096), *Physeter macrocephalus* (SWFSC Z12602), *Berardius arnuxii* (SWFSC Z9128), *Mesoplodon europaeus* (SWFSC Z11213), *Mesoplodon peruvianus* (SWFSC Z37886), *Pontoporia blainvillei* (SWFSC Z7351), *Inia geoffrensis* (SWFSC Z505), *Delphinapterus leucas* (SWFSC Z49102), *Neophocaena phocaenoides* (SWFSC Z9559), *Phocoena phocoena* (SWFSC Z23196), *Orcinus orca*, *Pseudorca crassidens* (SWFSC Z74710), *Feresa attenuata* (SWFSC Z18136), *Tursiops truncatus* (SWFSC Z126003), *Cephalorhynchus heavisidii* (SWFSC Z7320).

All data on body length were obtained from Slater *et al*.^10^, which compiled average adult female body length from many independent studies. Most data on body weight originated from Montgomery *et al*.^16^. This study took means of adult male and female values from multiple published sources. Data on several species including *B. musculus, C. marginata*, *N. phocaenoides*, *E. glacialis*, and *F. attenuata* are from Carwardine^5^. We took the average value of the adult body weight reported in Carwardine^5^ for these species. All phenotypic data are reported to two digits after the decimal point (Table 1).

### *MC4R* gene cloning and mutagenesis

We amplified 20 MC4R orthologs from the cetacean genomic DNA samples, and the MC4R sequence of *O. orca* was available from GenBank (accession number: XP_004268107). Primers were designed using the Primer Premier 5.0 software (Premier Biosoft International, Palo Alto, CA). Primers CeMC4Rfp (5′-GGTTAAGTCAATCCAGAG-3′) and CeMC4Rrp (5′-TGTGTTTAGCATCTCATCTG-3′), were designed based on the flanking sequences of the *MC4R* gene open reading frame (ORF) from *T. truncatus* reported in Ensembl. The PCR was performed using Phusion® High-Fidelity DNA Polymerase (New England Biolabs Inc.) with an optimized PCR program as follows: 30 s at 98 °C, followed by 40 cycles of denaturing at 98 °C for 30 s, annealing at 55 °C for 30 s, extension at 72 °C for 1 m, and a final 10 m extension at 72 °C in a Veriti 96 well thermal cycler (Applied Biosystem, CA, USA). The amplified PCR products were purified using QIAquick PCR purification kit (Qiagen, USA). The purified PCR products were sent to Genewiz Sequencing Company for sequencing with two primers (CeMC4Rfp and CeMC4Rrp), as well as two sequencing primers designed in the conserved region of the *MC4R* ORF. Final sequences were assembled by ChromasPro (Technelysium Ltd, Australia). Amino acid sequence alignment of MC4R was performed using Genedoc software (Free Software Foundation). The secondary structure of cetacean MC4R were determined by comparison to human and mouse MC4R.

To create expression constructs, *MC4R* was modified by adding an HA tag and a Kozak sequence to the N termini of the coding sequence and amplified by PCR using Phusion® High-Fidelity DNA Polymerase. The amplified PCR products were digested with appropriate restriction enzymes and subcloned into the pcDNA3.1(+) expression vector (Life Technologies, NY).

Point mutations in the *MC4R* gene were introduced with the Q5® Site-Directed Mutagenesis Kit (New England BioLabsInc, MA) and sequenced to confirm the substitutions. Primers for the human *MC4R* Q156R mutation were: 5′-CTATGCTCTCAGGTACCATAACATTAT-3′ and 5′-AAGATAGTAAAGTACCTGTCC-3′; and for the blue whale *MC4R* Q156R mutation were 5′-TTATGCCCTCAGGTACCATAACATC-3′ and 5′-AAGATCGTAAAATACCTGTC-3′.

### MC4R evolutionary and phylogenetic analysis

Coding sequences of *MC4R* orthologs from 12 additional Laurasiatherian mammals were retrieved from the ENSEMBL database (version 83) as outgroups: *Ailuropoda melanoleuca* (ENSAMEG00000019205), *Bos taurus* (ENSBTAG00000019676), *Canis familiaris* (ENSCAFG00000000090), *Equus caballus* (ENSECAG00000001712), *Erinaceus europaeus* (ENSEEUG00000011663), *Felis catus* (ENSFCAG00000006540), *Mustela putorius* (ENSMPUG00000019609), *Myotis lucifugus* (ENSMLUG00000000218), *Ovis aries* (ENSOARG00000004034), *Pteropus vampyrus* (ENSPVAG00000004246), *Sus scrofa* (ENSSSCG00000004904), and *Vicugna pacos* (ENSVPAG00000009923). The coding sequences of all 33 *MC4R* genes were aligned using PRANK v150803 with the codon model^45^. Identical alignments were obtained using alternative alignment strategies, including translated protein sequence based alignment using either PRANK with the protein model or MAFFT v7.158^46^ with the “G-INS-i” algorithm. Four different phylogenetic analyses were conducted to infer the evolutionary history of MC4R. Firstly, a codon-based maximum-likelihood (ML) tree was estimated using IQTREE v1.4.1^47^ with the semi-empirical model “MGK + SCHN05 + F3X4 + G4” which was found to be the best-fitting model using the model selection function of IQTREE. The number of unsuccessful iterations before stop was set to 500 and the approximate Bayes test was used to evaluate the reliability of the result. A second codon-based ML analysis was performed using CodonPhyMLv1.00^48^ with the BIONJ tree and five random trees as starting trees, the best of NNIs and SPRs as the search strategy, and the number of gamma categories set to three. Similar to the IQTREE analysis, the semi-empirical model “MGECMS05” was used. The third ML phylogenetic analysis was based on the nucleotide alignment and carried out using RAxML v8.2.0^49^ with 1000 replicates of rapid bootstrap searches followed by a search for the best tree in the same run. The model “GTRGAMMA” was found to be the best-fitting model among the three models supported by RAxML. Lastly, a Bayesian analysis of the nucleotide alignment was conducted using MrBayes v3.2.6^50^ with the “mixed” model. The analysis consisted of two parallel runs (each with four chains) and converged after one million generations. 10,000 trees were sampled (one from every 100 generations) and summarized into a consensus tree after discarding the first 2,500 sampled trees as burn-in.

The Approximately Unbiased (AU) test^51^ was used to compare the estimated gene phylogenies of *MC4R* and the previously reported species phylogeny of Cetacea^3^. Each tree topology was analyzed by IQTREE under the best-fitting codon model to optimize branch-lengths and model parameters. Per-site likelihood values were calculated accordingly and used for the AU-test by CONSEL v0.20^52^. A second AU-test was performed on the same set of gene/species tree topologies based on the nucleotide alignment under the best-fitting DNA model.

To investigate the selective pressure during the evolution of cetacean *MC4R* genes, the overall ratio of non-synonymous and synonymous substitution rates (ω ratio) for cetacean *MC4R* genes was estimated using the CODEML program (M0 model) in the PAML v4.8 package^53^. In addition, we tested the hypothesis that the *MC4R* gene has experienced positive selection in a subset of sites in the most recent common ancestor (MRCA) of Odontoceti. In the test, the internal branch leading to the most recent common ancestor of Odontoceti was selected as the foreground and a branch-site test was conducted using CODEML. The modified model A where a subset of sites were under positive selection on the foreground branch but under neutral or purifying selection elsewhere was compared with the null model where no sites were under positive selection. All analyses were performed with three different starting values of ω (i.e. 0.1, 1.0, 1.5) to avoid local optima. The statistical significance was calculated using the likelihood ratio test.

### Cell culture and functional assays

Human embryonic kidney (HEK) 293T cells were purchased from American Type Culture Collection (Manassas, VA), and were grown in modified Eagle’s medium containing 10% fetal bovine serum, 100 units/ml penicillin and 100 μg/ml streptomycin at 37 °C in a humidified 5% CO_2_ incubator. Cells were transfected with the appropriate plasmids with LipoD293 (SignaGen Laboratories, MD) approximately 36–48 h before measuring ligand binding and signaling.

#### cAMP accumulation assays for MC4R activity

HEK293T cells were plated in 10 cm dishes, transfected with CRE-luciferase reporter plasmid and indicated plasmids, and reseeded onto 96-well or 384-well plates 36–48 h later after transfection. Cells were challenged with vehicle or ligand in MEM supplemented with 0.1% bovine serum albumin (BSA) for 4 h at 37 °C. Luciferase activity was measured 10 minutes after addition with One-Glo luciferase assay reagent (Promega Corp., WI) in a Spectramax M5 reader (Molecular Devices, CA). Nonlinear regression analysis was performed using GraphPad Prism software (GraphPad Software Inc., CA).

#### Competitive displacement binding assay

HEK293T cells were transiently transfected and seeded as described above *(1)* in 96 well plates. Cells were incubated for 20 min in binding buffer [Dulbecco’s phosphate-buffered saline (DPBS, Invitrogen, CA) containing 20 mM HEPES (pH7.5), and 0.1% BSA at 37 °C, and were then incubated for 1 hour in binding buffer (50 ml per well) with 2 nM of Eu-labeled NDP-α-MSH (Perkin Elmer, MA) and in indicated concentration of unlabeled NDP-α-MSH (Fig. 5c). Dishes were placed on ice and washed three times with cold PBS; 50 ml DELPHIA enhancing solution was then added and fluorescence was measured 30 min to 1 h later on a Spectramax M5 reader.

#### Constitutive activity assay

Mutant and wild-type plasmid DNAs were transfected simultaneously at different concentrations as described above. Empty vector pcDNA3.1(+) was used to normalize the amount of plasmid DNA added to each well. 36 to 48 h after transfection, the cells were loaded on the 96-well plates and phosphodiesterase inhibitor IBMX added before incubating for 4 h. The CRE-luciferase reporter gene bioassays were performed as described above; only basal and non-receptor dependent forskolin values were measured. The average of six data points at each concentration was determined in three independent experiments. Concentration of DNA used in the transient transfection bioassay is shown in Table 3.

**Table 3:** DNA concentrations used in constitutive activity assay (Fig. 5d).

conc plotted (ng)	0	1.25	6.25	12.5	18.75	25
pCDNA3.1 (+)-MC4R	0	1.25	6.25	12.5	18.75	25
pCDNA3.1 (+)	25	23.75	18.75	12.5	6.25	0
CRE-luciferase	0.975	0.975	0.975	0.975	0.975	0.975

The correlations between MC4R activity (EC_50_ value) and phenotypic data (body weight and body length) in cetaceans were calculated using Bayes Traits v2.0 which properly accounts for the phylogenetic relationships between the organisms we sampled in this study. Body weight data were log-transformed so that they fit a normal distribution. Using the previously reported cetacean phylogeny^3^ as guide tree, maximum likelihood values were calculated with and without assuming a correlation between MC4R activity and phenotypic data under the random walk model. The difference between the two likelihood values was used to determine statistical significance using the likelihood ratio test (degree of freedom: 1).

### Ethics Statement

The cetacean DNA samples for this study were provided by K. Robertson from an extant repository of the Southwest Fisheries Science Center, as authorized under a marine mammal permit issued by NOAA Fisheries.

### Data availability

DNA sequences generated as part of this study have been submitted to GenBank (accession numbers: KM385448-KM385467).

## Electronic supplementary material

Supplementary Dataset
