# Supplementary material for: Functional variants of the melanocortin-4 receptor associated with the Odontoceti and Mysticeti suborders of cetaceans
Source: Sci Rep. 2017 Jul 18;7:5684. doi: 10.1038/s41598-017-05962-1 (PMC5515947; doi:10.1038/s41598-017-05962-1)
Supplement: Supplementary file 1 — Supplementary Dataset [file 41598_2017_5962_MOESM1_ESM.docx]

**Functional variants of the melanocortin-4 receptor associated with the Odontoceti and Mysticeti suborders of cetaceans**

**Liyuan Zhao^1,2^, Xiaofan Zhou^3,5^, Antonis Rokas^3^, and Roger. D. Cone^*,4^**

**SUPPLEMENTARY DATA**

**Supplementary Table 1:** Results of the AU test between the reference phylogeny reflecting species relationships between the 33 species[^1^](#_ENREF_1), and *MC4R* gene phylogenies recovered using four different phylogenetic approaches.

| 1. The AU-test based on codon alignment^a^ | | | |
| --- | --- | --- | --- |
| Rank | Tree name | ΔlnL | p-value of AU test |
| 1 | Codonphyml | 0 | 0.634 |
| 2 | IQTREE | 0.1 | 0.644 |
| 3 | Reference phylogeny | 18.1 | 0.073 |
| 1. AU-test based on nucleotide alignment^b^ | | | |
| Rank | Tree name | deltaLoglk | p-value of AU test |
| 1 | RAxML | 0 | 0.933 |
| 2 | MrBayes | 11.8 | 0.113 |
| 3 | Reference phylogeny | 11.8 | 0.137 |

^a^sitewise log likelihood values were calculated based on the codon alignment by CodonPhyML under the semi-empirical model “MGECMS05”;

^b^sitewise log likelihood values were calculated based on the nucleotide alignment by RAxML under the “GTRGAMMA” model.

S**upplementary Table 2:** Results of branch-site test of positive selection.

| Model | ω_0_ / *p*_0_ | ω_1_ / *p*_1_ | ω_2(foreground)_ / *p*_2a_ | ω_2(foreground)_ / *p*_2b_ | 2ΔlnL / df | *p*-value of χ^2^ test |
| --- | --- | --- | --- | --- | --- | --- |
| null model | 0.048 / 74.72% | 1 / 4.00% | 1 / 20.20% | 1 / 1.08% | 0.20 / 1 | 0.655 |
| model A^a^ | 0.048 / 94.55% | 1 / 5.06% | 117.00 / 0.37% | 117.00 / 0.02% |  |  |

^a^In model A, the branch leading to the Odontoceti clade was selected as the foreground whereas all the other branches were selected as background.

In this branch-site test, the sites are divided into four categories. Sites in the first two categories are under the same selective pressure (less than one for the first category [ω0] and equal to one for the second category [ω1]) on both the background and foreground branches. In contrast, sites in the other two categories are under the different selective pressures (ω0 and ω1 on the background branches, respectively, and ω2 on the foreground branches) between background and foreground branches. The fractions of sites in the four categories are p0, p1, p2a, and p2b, respectively. ω2 is no less than 1 in the model A while it is restricted to 1 in the null model.


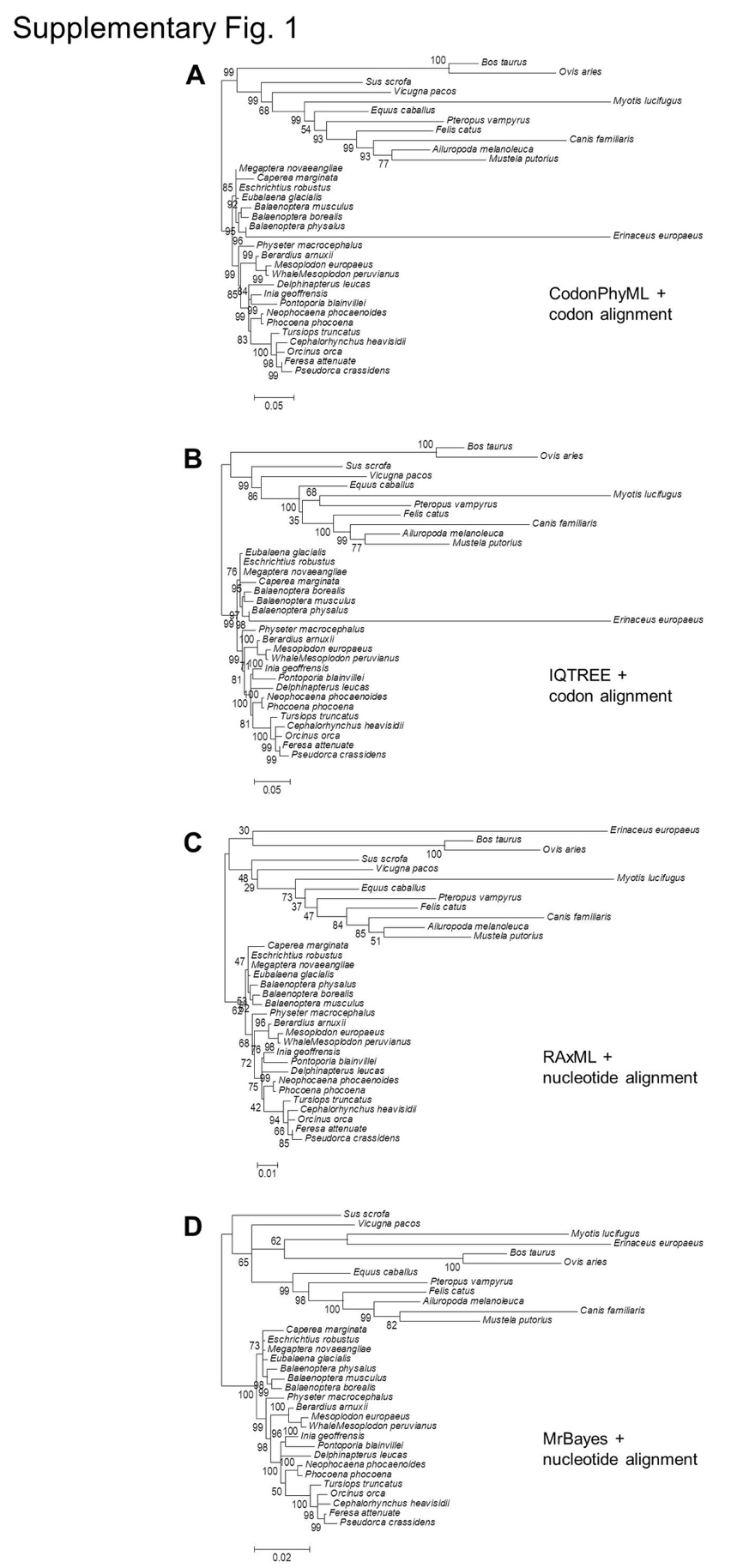


**Supplementary Figure 1:** Phylogenetic analyses of *MC4R* from 21 cetacean species with 12 other Laurasiatherian mammals as outgroups, using the indicated approaches and model types.

**References**

1 Steeman, M. E. *et al.* Radiation of extant cetaceans driven by restructuring of the oceans. *Systematic biology* **58**, 573-585, (2009).
